# Predicting the Transition to Metabolically Unhealthy Obesity Among Young Adults With Metabolically Healthy Obesity in South Korea: Nationwide Population-Based Study

**DOI:** 10.2196/52103

**Published:** 2024-06-28

**Authors:** HyunHae Lee, Ji-Su Kim, Hyerine Shin

**Affiliations:** 1 School of Nursing University of Washington Seattle, WA United States; 2 Department of Nursing Chung-Ang University Seoul Republic of Korea

**Keywords:** metabolically healthy obesity, metabolic syndrome, metabolically unhealthy obesity, nomogram, obesity, young adult, male, noncommunicable disease, South Korea, population-based study, intervention

## Abstract

**Background:**

Globally, over 39% of individuals are obese. Metabolic syndrome, usually accompanied by obesity, is regarded as a major contributor to noncommunicable diseases. Given this relationship, the concepts of metabolically healthy and unhealthy obesity, considering metabolic status, have been evolving. Attention is being directed to metabolically healthy people with obesity who have relatively low transition rates to noncommunicable diseases. As obesity rates continue to rise and unhealthy behaviors prevail among young adults, there is a growing need for obesity management that considers these metabolic statuses. A nomogram can be used as an effective tool to predict the risk of transitioning to metabolically unhealthy obesity from a metabolically healthy status.

**Objective:**

The study aimed to identify demographic factors, health behaviors, and 5 metabolic statuses related to the transition from metabolically healthy obesity to unhealthy obesity among people aged between 20 and 44 years and to develop a screening tool to predict this transition.

**Methods:**

This secondary analysis study used national health data from the National Health Insurance System in South Korea. We analyzed the customized data using SAS (SAS Institute Inc) and conducted logistic regression to identify factors related to the transition from metabolically healthy to unhealthy obesity. A nomogram was developed to predict the transition using the identified factors.

**Results:**

Among 3,351,989 people, there was a significant association between the transition from metabolically healthy to unhealthy obesity and general characteristics, health behaviors, and metabolic components. Male participants showed a 1.30 higher odds ratio for transitioning to metabolically unhealthy obesity than female participants, and people in the lowest economic status were also at risk for the transition (odds ratio 1.08, 95% CI 1.05-1.1). Smoking status, consuming >30 g of alcohol, and insufficient regular exercise were negatively associated with the transition. Each relevant variable was assigned a point value. When the nomogram total points reached 295, the shift from metabolically healthy to unhealthy obesity had a prediction rate of >50%.

**Conclusions:**

This study identified key factors for young adults transitioning from healthy to unhealthy obesity, creating a predictive nomogram. This nomogram, including triglycerides, waist circumference, high-density lipoprotein-cholesterol, blood pressure, and fasting glucose, allows easy assessment of obesity risk even for the general population. This tool simplifies predictions amid rising obesity rates and interventions.

## Introduction

In the last 40 years, the number of people with obesity has more than tripled worldwide; as of 2016, 39% of the global population was estimated to have a BMI ≥25 kg/m^2^ [1]. Obesity, usually accompanied by metabolic syndrome (MetS), is a major risk factor for noncommunicable diseases (NCDs), such as cancer, musculoskeletal problems, and cardiovascular diseases [2]. Epidemiological studies showed that 20%-45% of people have MetS. Furthermore, MetS prevalence is anticipated to increase to >50% by 2035 [3].

Recent studies have increasingly emphasized metabolically healthy obesity (MHO) and metabolically unhealthy obesity (MUO), with rising interest in the connection between obesity and MetS [4-6]. Studies revealed that individuals with MHO have a significantly reduced incidence of common obesity-related disorders, such as stroke, cancer, cardiovascular disease, and type 2 diabetes [7]. However, recent research indicates that individuals with MHO might eventually transition to MUO [6], with up to 50% of the population with obesity possibly falling into the MHO category [8]. This underlines the need for research and treatment to prevent or interrupt the transition from MHO to MUO.

On the other hand, young adults face higher mortality from obesity than older people. As social relationships that foster independence evolve, the prevalence of unhealthy lifestyle behaviors, such as smoking, alcohol consumption, and physical inactivity, has been rapidly increasing, contributing to chronic diseases in the population with obesity [9,10]. Additionally, the COVID-19 pandemic caused increased psychological stress, decreased physical activity, and altered eating patterns in young adults, contributing to obesity [11]. Young adults are more aware of obesity than older populations but engage less in weight loss programs and achieve less intentional weight loss [12], underscoring the need for proactive interventions and tailored programs for this age group.

This study identified risk factors and developed a screening tool with a nomogram to prevent the transition from MHO to MUO. A nomogram, a graphical tool that can perform a complicated approximation calculation, provided a clear interpretation of which predictors could be more critical factors [13]. The use of this scale also allows the general population to intuitively and easily assess the likelihood of transitioning to MUO. Therefore, this study aimed to predict the transition from MHO to MUO among young individuals with obesity using the nomogram.

## Methods

### Study Setting

The Korean government provides health checkups for 97% of the South Korean population through public health insurance, while the remaining 3% receive these checkups through medical aid programs. Residents are encouraged to undergo biennial health checkups, covering measurements such as height, weight, BMI, blood tests, and urinary tests. The National Health Insurance System (NHIS) is tasked with collecting medical records, insurance statuses, and other pertinent data through these enrollment methods, and providing corresponding services [14].

### Measures

MHO and MUO are defined based on the metabolic status of the population with obesity. Obesity is characterized by an abnormal or excessive accumulation of adipose tissue, and BMI is primarily used to define it [1]. According to evidence regarding the high risk of type 2 diabetes or cardiovascular diseases with lower BMI standards, the World Health Organization (WHO) proposed each country make decisions about obesity definitions [15,16]. The Korean Society for the Study of Obesity defined obesity as a BMI (weight in kg divided by the square of height in meters) ≥25 kg/m^2^ [17]. Indicators for assessing metabolic status include waist circumference, fasting blood glucose levels, blood pressure, high-density lipoprotein (HDL) cholesterol levels, and fasting triglyceride levels. This study used the cutoff points ≥90 cm in male participants and ≥85 cm in female participants for waist circumference (a Korean-specific cutoff point) [17]; ≥130/85 mmHg or using antihypertensive medications for blood pressure; ≥100 mg/dL or using antidiabetic medications for fasting blood glucose; ≥150 mg/dL for fasting triglycerides; and <40 mg/dL in male participants and <50 mg/dL in female participants for HDL cholesterol [18]. An individual is defined as metabolically unhealthy if ≥3 of these 5 indicators yield abnormal results. Therefore, MUO is a state of obesity with 3 or more abnormal indicators of metabolic status. Those who do not meet these criteria are defined as MHO [5].

In this study, healthy behaviors (smoking, alcohol consumption, and regular exercise), which are representative factors influencing MetS and obesity, were examined. Participants were classified according to their smoking status. Alcohol consumption was assessed by categorizing participants into individuals who consume alcohol heavily (heavy; ≥30 g/day), moderate alcohol (moderate; <30 g/day), and do not drink alcohol (none) [19]. Regular exercise was defined as engaging in high-intensity exercise for at least 20 minutes, ≥3 times per week, or moderate-intensity exercise for at least 30 minutes, ≥5 times per week [20].

### Study Population

We identified participants (aged ≥20 years) who had health checkups in 2009-2010 and 2013-2014. A total of 3,351,989 individuals were obese (BMI ≥25 kg/m^2^) during both examinations. From this group, we excluded those who had MUO during the first examination and selected those aged between 20 and 44 years during the second examination. We also excluded individuals diagnosed with cancer or cardiovascular diseases (myocardial infarction and stroke) before the second examination. After excluding missing values, the final sample size included in the analysis was determined to be 562,765 individuals.

### Data Analysis

Young adults’ general characteristics, health behaviors, and metabolic statuses were summarized using the chi-square test and 2-tailed *t* tests. We used logistic regression analysis to examine the association between the transition to MUO and general characteristics, health behaviors, and metabolic states. Based on the results of the logistic regression, factors associated with the transition to MUO were identified, and scores were assigned ranging from 0 to 100. The prediction model for calculating the risk of the transition to MUO was depicted using a nomogram. *P* values <.05 were considered statistically significant. Statistical analysis was conducted using SAS (version 9.4; SAS Institute Inc).

### Ethical Considerations

The project was submitted for evaluation to the institutional review board of Chung-Ang University (1041078-202203-HR-080). Since the study was a secondary analysis using the NHIS data, the institutional review board exempted the study from formal ethical approval. All participants who underwent health checkups through NHIS consented to provide the results and answers for research purposes. The data were deidentified by the NHIS and provided with permission.

## Results

### General Characteristics and Metabolic Status of Participants According to Metabolic Status After 4 Years

The general characteristics of participants who transitioned from MHO to MUO over a 4-year period, compared to those who did not, are outlined in Table 1. A higher proportion of male participants was observed in the group that transitioned to MUO, with a statistically significant difference (*P*<.001). Age, income level, and health behaviors (smoking status, alcohol consumption, and regular exercise) showed statistically significant differences as well (*P*<.001). Both groups showed significant differences regarding metabolic status based on the screening time point, even at the initial screening. Mainly, the group that transitioned to MUO exhibited remarkable changes in metabolic status, with variations in triglyceride, fasting glucose, waist circumference, blood pressure, and HDL.

**Table 1:** General characteristics of participants according to metabolic status after 4 years.

Characteristics		Total (N=562,765)	Metabolically healthy obesity (n=391,593)	Metabolically unhealthy obesity (n=171,172)	*P* value	
**General characteristics**						<.001
	Sex (male), n (%)	460,022 (81.74)	311,859 (79.64)	148,163 (86.56)		
	Age (years), mean (SD)	37.39 (4.87)	37.34 (4.91)	37.52 (4.78)		
	Income (Q1^a^), n (%)	45,302 (8.05)	32,297 (8.25)	13,005 (7.6)		
**Smoking status, n (%)**						<.001
	Never	224,106 (39.82)	167,087 (42.67)	57,019 (33.31)		
	Past	112,365 (19.97)	77,603 (19.82)	34,762 (20.31)		
	Current	226,294 (40.21)	146,903 (37.51)	79,391 (46.38)		
**Alcohol consumption, n (%)**						<.001
	None	166,112 (29.52)	120,089 (30.67)	46,023 (26.89)		
	Moderate	329,432 (58.54)	229,660 (58.65)	99,772 (58.29)		
	Heavy	67,221 (11.94)	41,844 (10.69)	25,377 (14.83)		
Regular exercise (yes), n (%)		110,662 (19.66)	83,001 (21.2)	27,661 (16.16)	<.001	
BMI (kg/m^2^), mean (SD)		27.77 (2.33)	27.36 (2.05)	28.71 (2.65)	<.001	
Waist circumference (cm), mean (SD)		88.94 (7.04)	87.34 (6.55)	92.61 (6.75)	<.001	
Fasting glucose (mg/dL), mean (SD)		96.02 (16.79)	92.83 (12.5)	103.32 (22.21)	<.001	
Systolic blood pressure (mm Hg), mean (SD)		124.1 (12.52)	121.48 (11.67)	130.08 (12.35)	<.001	
Diastolic blood pressure (mm Hg), mean (SD)		78.4 (9.34)	76.64 (8.73)	82.43 (9.46)	<.001	
HDL^b^-cholesterol (mg/dL), mean (SD)		51.04 (13.6)	53.04 (13.64)	46.45 (12.32)	<.001	
Triglycerides (mg/dL), geometric mean (95% CI)		134.9 (134.7-135.1)	115.6 (115.4-115.8)	192.1 (191.7-192.6)	<.001	
**Metabolic syndrome component (initial exam), n (%)**						<.001
	Abnormal waist circumference	167,920 (29.84)	104,932 (26.8)	62,988 (36.8)		
	Abnormal fasting glucose	85,539 (15.2)	56,577 (14.45)	28,962 (16.92)		
	Abnormal blood pressure	180,849 (32.14)	117,449 (29.99)	63,400 (37.04)		
	Abnormal HDL-cholesterol	79,524 (14.13)	51,530 (13.16)	27,994 (16.35)		
	Abnormal triglycerides	167,215 (29.71)	99,100 (25.31)	68,115 (39.79)		
**Metabolic syndrome component (after 4 years), n (%)**						<.001
	Abnormal waist circumference	268,350 (47.68)	136,694 (34.91)	131,656 (76.91)		
	Abnormal fasting glucose	168,896 (30.01)	70,083 (17.9)	98,813 (57.73)		
	Abnormal blood pressure	246,816 (43.86)	120,013 (30.65)	126,803 (74.08)		
	Abnormal HDL-cholesterol	136,472 (24.25)	53,000 (13.53)	83,472 (48.76)		
	Abnormal triglycerides	242,884 (43.16)	104,619 (26.72)	138,265 (80.78)		

^a^Participants who got medical aid or was included in the lowest quartile of income.

^b^HDL: high-density lipoprotein.

### Logistic Regression Analyses of the Transition From MHO to MUO

We identified factors contributing to the transition from MHO to MUO through logistic regression analysis (Table 2). Adjusting for all significant characteristics identified in Table 1, we constructed model 2. The model demonstrated that transitioning from MHO to MUO was independently associated with an increase in age.

**Table 2:** Logistic regression analysis of the transition from metabolically healthy obesity to metabolically unhealthy obesity.

Variables			Participants, n	Metabolically unhealthy obesity, n (%)	Model 1, odds ratio (95% CI)^a^	Model 2, odds ratio (95% CI)^a^
**General characteristics**						
	Age (per 1 year)		—^b^	—	1.008 (1.006-1.009)	1.004 (1.002-1.005)
	**Sex**					
		Male	460,022	148,163 (32.21)	1.65 (1.62-1.67)	1.3 (1.27-1.32)
		Female	102,743	23,009 (22.39)	1 (Reference)	1 (Reference)
	**Income^c^**					
		Q1	45,302	13,005 (28.71)	0.92 (0.9-0.93)	1.08 (1.05-1.1)
		Q2-Q4	517,463	158,167 (30.57)	1 (Reference)	1 (Reference)
	**Smoking status**					
		Never	224,106	57,019 (25.44)	1 (Reference)	1 (Reference)
		Past	112,365	34,762 (30.94	1.31 (1.29-1.33)	1.1 (1.09-1.12)
		Current	226,294	79,391 (35.08)	1.58 (1.56-1.6)	1.27 (1.25-1.29)
	**Alcohol consumption**					
		None	166,112	46,023 (27.71)	1 (Reference)	1 (Reference)
		Moderate	329,432	99,772 (30.29)	1.13 (1.12-1.15)	0.99 (0.98-1.01)
		Heavy	67,221	25,377 (37.75)	1.58 (1.55-1.61)	1.26 (1.23-1.28)
	**Regular exercise**					
		No	452,103	143,511 (31.74)	1 (Reference)	1 (Reference)
		Yes	110,662	27,661 (25)	0.72 (0.71-0.73)	0.72 (0.7-0.73)
**Metabolic syndrome component (first exam)**						
	**Waist circumstance**					
		No	394,845	108,184 (27.40)	1 (Reference)	1 (Reference)
		Yes	167,920	62,988 (37.51)	1.59 (1.57-1.61)	2.11 (2.08-2.14)
	**Fasting glucose**					
		No	477,226	142,210 (29.8)	1 (Reference)	1 (Reference)
		Yes	85,539	28,962 (33.86)	1.21 (1.19-1.23)	1.69 (1.66-1.72)
	**Blood pressure**					
		No	381,916	107,772 (28.22)	1 (Reference)	1 (Reference)
		Yes	180,849	63,400 (35.06)	1.37 (1.36-1.39)	1.76 (1.74-1.78)
	**HDL^d^-cholesterol**					
		No	483,241	143,178 (29.63)	1 (Reference)	1 (Reference)
		Yes	79,524	27,994 (35.2)	1.29 (1.27-1.311)	1.89 (1.86-1.92)
	**Triglycerides**					
		No	395,550	103,057 (26.05)	1 (Reference)	1 (Reference)
		Yes	167,215	68,115 (40.73)	1.95 (1.93-1.98)	2.25 (2.22-2.28)

^a^Model 1 was not adjusted, and model 2 was adjusted for age, sex, income, smoking, drinking, regular exercise, waist circumstance, fasting glucose, blood pressure, HDL-cholesterol, and triglycerides; all variables had a statistically significant association with the transition to metabolically unhealthy obesity (*P*<.001).

^b^Not applicable.

^c^Q1-Q4, first through fourth quartiles.

^d^HDL: high-density lipoprotein.

For each additional year of age, the odds ratio (OR) of transitioning to MUO was 1.04 times higher (95% CI 1.002-1.005). Male participants showed a 1.30 times higher OR of transitioning to MUO than female participants (95% CI 1.27-1.32). People belonging to the lowest economic group showed a 1.08 higher OR of transitioning to MUO than other economic status groups (95% CI 1.05-1.1). Regarding healthy behaviors, having a history of smoking and being an individual who currently smoke showed 1.1 (95% CI 1.09-1.12) and 1.27 (95% CI 1.25-1.29) higher OR of transitioning to MUO than people without a smoking history. Individuals consuming alcohol heavily had a 1.26 higher OR (95% CI 1.23-1.28) of transitioning to MUO compared to people not drinking alcohol. People participating in regular exercise had a 0.72 lower OR (95% CI 0.7-0.73) of transitioning to MUO than people not regularly exercising. Abnormal waist circumference at the initial screening (OR 2.11, 95% CI 2.08-2.14), impaired fasting glucose or related medication use (OR 1.69), hypertension or related medication use (OR 1.76, 95% CI 1.76-1.78), low HDL cholesterol or related medication use (OR 1.89, 95% CI 1.86-1.92), and elevated triglyceride or related medication use (OR 2.25, 95% CI 2.22-2.28) were all statistically significantly associated with the transition to MUO.

### Prediction of the Transition From MHO to MUO

**Figure 1:**
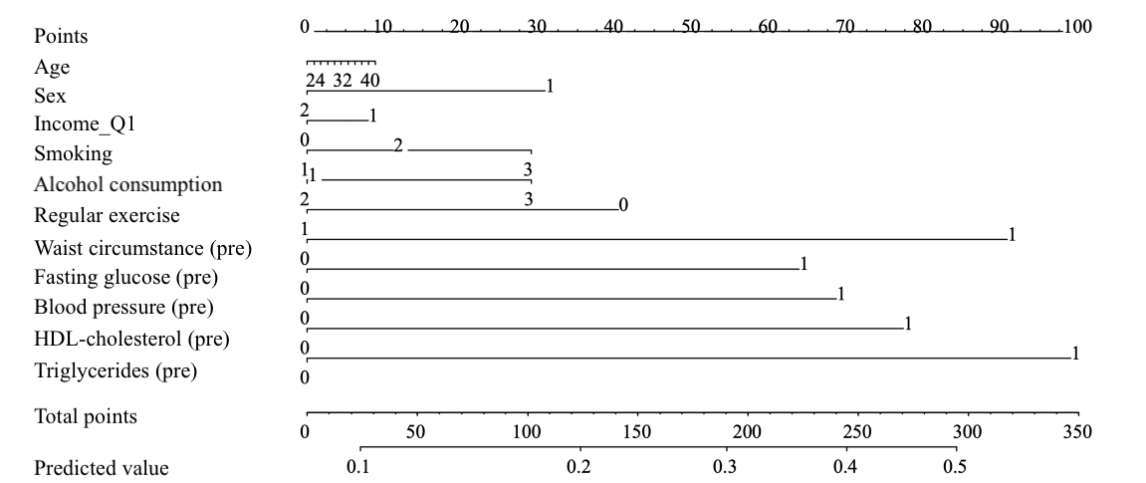
The nomogram for predicting the transition from metabolically healthy obesity to metabolically unhealthy obesity.

**Figure 2:**
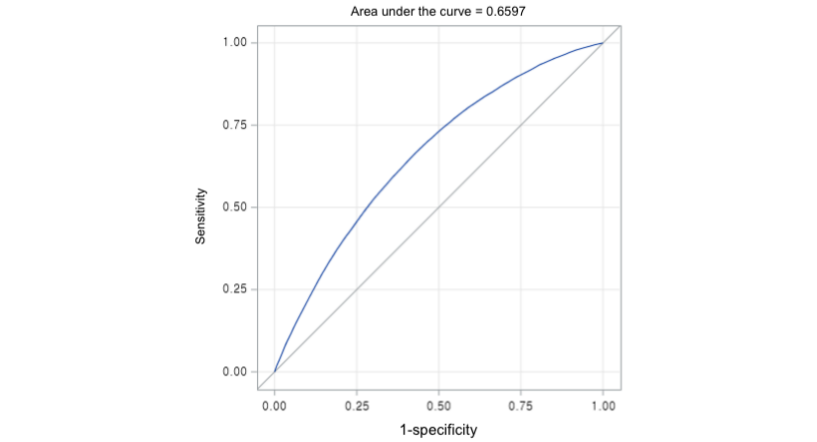
A receiver operating characteristic (ROC) curve of the nomogram (model 2).

**Figure 3:**
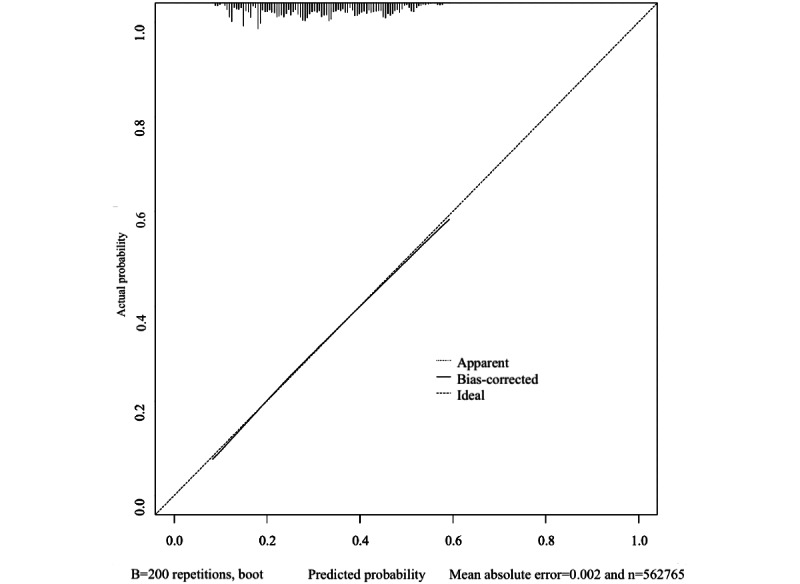
The calibration curves for the nomogram for predicting the transition from metabolically healthy obesity to metabolically unhealthy obesity. The dotted line represents the ideal concordance between the predicted and actual probabilities. The solid line represents the performance of the nomogram.

A nomogram (Figure 1) was constructed to assist clinical predictions, providing a quantitative estimation of the probability of transitioning to MUO. This is a screening tool for predicting the transition from MHO to MUO with 66% discriminative ability (Figure 2). According to individual characteristics, each value is located based on each variable in the nomogram. At each variable point, a vertical line is drawn up to the scale of “points,” followed by identifying the point of the variable. After marking all values based on variables, all points of each variable are summarized, and the point on the scale of “total points” is marked. At the total point, after drawing a vertical line to the scale of “predicted value,” the matched point will be the individual’s predicted value of transitioning from MHO to MUO. Using multivariate logistic regression, we identified 11 independent predictors, assigning the highest score in the nomogram to each variable (eg, 44 years: 9 points; male participant: 32 points; first income level quartile: 9 points; individual who smokes: 29 points; individual who drinks: 29 points; no regular physical activity: 41 points; abnormal waist circumference: 92 points; abnormal fasting glucose or taking diabetes medications: 65 points; abnormal blood pressure or taking hypertension medication: 70 points; abnormal HDL level or taking dyslipidemia medications: 78 points; abnormal serum triglyceride level or taking dyslipidemia medication: 100 points; and total points: 344 points). When the total points reached 295, the prediction rate for transitioning from MHO to MUO was determined to be higher than 50%. As depicted in Figure 3, the nomogram showed close agreement between predicted and observed outcomes in the study population, with a slight tendency for overestimation in the 0.1-0.2 and 0.5-0.6 intervals. Figure 2 shows the receiver operating characteristic curve used to validate the nomogram, yielding an area under the curve of 0.66 (95% CI 0.658-0.661).

## Discussion

### Principal Findings

The prevalence of obesity is escalating globally, accompanied by MetS, increasing the risk of NCDs. This upward trend among young adults can be attributed to lifestyle alterations during adulthood [10] and, more recently, decreased physical activity or changed dietary habits due to the COVID-19 pandemic [11]. Moreover, individuals with MUO face substantially elevated risks of NCDs compared to those with MHO [4]. Considering that MHO might merely represent a “honeymoon phase” preceding the shift to MUO [6], the urgency of managing MHO among young adults is underscored.

This study investigated the influence of sociodemographic factors, metabolic status, and health behaviors on MetS development. By comparing young adults who transitioned from MHO to MUO with those who maintained their health status, we identified associations between the transition and factors with sex, low socioeconomic status, 5 metabolic variables, and health-related behaviors (drinking, smoking, and irregular physical activity). The nomogram was constructed to predict the transition from MHO to MUO using the data from the results.

Transitioning to MUO was higher in male participants than in female participants. In a previous study, MetS prevalence based on sex showed various results according to age. A cross-sectional study using data from the Third National Health and Nutrition Examination Survey in America showed a similar result to this study: a higher prevalence of MetS in male individuals [21]. However, they identified that women showed a higher MetS prevalence than men in older age. Using a similar perspective, a cohort study from Switzerland reported that the sex-based prevalence of MetS could be different according to the age range [22]. In Korea, the MetS fact sheet for 2010 using data from the Korean National Health and Nutrition Examination Surveys from 2007 to 2018 reported a higher prevalence in the male population but increasing trends in the female population aged >70 years [23]. Thus, our participants were constrained to young adults. Additionally, the results of this study were similar to those of previous studies. Regarding economic status, individuals with lower income levels showed a compatibly higher risk of transitioning to MUO, aligning with previous studies on risk factors for MetS development [24,25]. Our findings align with the reality that obesity is more prevalent than underweight, especially in low-income countries [1]. Furthermore, dietary issues in these regions correlate with diet-related NCDs [1]. Consequently, the findings emphasize the need to consider sex and the importance of health care provision for those in low socioeconomic positions within the community in the management of individuals with MHO to prevent transitioning to MUO.

The associations between obesity and health behaviors, that is, smoking status [26], alcohol consumption [27], and physical activity [28], have been widely investigated and linked to MetS [29]. This study further validated the significance of health-related behavior patterns in the transition from MHO to MUO in young adults with obesity. We found that moderate alcohol consumption (<30 g of alcohol daily) was associated with a decreased transition to MUO. Similar to the result, previous research [30] indicated a positive correlation between moderate alcohol consumption and a reduced risk of MetS. However, a study targeting Asian populations indicated that even moderate alcohol intake might affect metabolic status [31]. Recognizing that alcohol consumption itself could affect dietary habits in the population with obesity, as well as the possibility that the frequency of alcohol consumption might influence the occurrence of MetS more than the quantity consumed, is crucial [32]. Therefore, a particular caution, including restricting the frequency of consumption, is required regarding alcohol consumption to prevent the onset of MetS in patients with obesity.

We found that abnormal metabolic factors played a crucial role in the transition to MUO over a 4-year span. Specifically, abnormal waist circumference and triglyceride levels showed nearly ≥2 higher ORs for the transition to MUO. Waist circumference was recognized as a more pertinent obesity measure than BMI [33] and is frequently used to forecast cardiometabolic disorders [34]. Waist circumference is strongly linked to abnormal fasting glucose [35] and lipid profiles [36], both affecting metabolic states. Our findings underscore the importance of waist circumference control by demonstrating its association with the development of MUO, with an OR >1.6 for other abnormal metabolic variables. Unlike other metabolic conditions that might demand pharmacological management, waist circumference is a physical attribute that can be reduced through lifestyle changes, such as exercise, dietary adjustments, and curbed alcohol consumption [37]. Managing waist circumference is not only a strategy for combating obesity but also for improving other abnormal metabolic disorders [37]. Therefore, tailored interventions aimed at trimming waist circumference are essential. Additionally, our findings revealed a pronounced association between triglyceride levels and the transition to MUO, connecting high triglyceride levels with prevalent issues such as type 2 diabetes and abnormal HDL in people with obesity [38]. Approximately 15 years ago, a cross-sectional study in America revealed that abnormal triglyceride and HDL levels were one of the most prevalent MetS combination factors in young male participants and female participants [39]. Still, the research even proposed triglyceride levels and waist circumference as specific indicators for predicting MetS in African American people [40]. For individuals with MHO with abnormal metabolic factors, proactive management, possibly including medication control and lifestyle modifications, is vital. More intensive interventions might be warranted in abnormal waist circumference and serum triglyceride cases.

The nomogram was developed with identified factors, predicting the transition from MHO to MUO. As a simplified tool [13], the nomogram allows young adults with MHO to directly calculate their risk of transitioning to MUO with their health examination results. Created using data from the NHIS, it can serve as a valuable resource for adults undergoing regular health checkups, enabling them to assess their risk and formulate personalized health plans. Individuals can easily evaluate their risks without relying on health care professionals or medical experts. People with MHO can efficiently use this tool to tailor their interventions by entering their sex, health behaviors, and metabolic status scores into the nomogram.

### Limitations

The study had some limitations as a secondary analysis of Korean health examination data. Although dietary habits or nutrition consumption are critical factors for obesity or MetS, we could not include them as variables since we used publicized NHIS data. Second, while clinical measurements were accurately performed in a hospital setting, health behavior results—obtained through self-response surveys—might be subjective. Third, further validation for other racial groups is needed. Differences in obesity [41] and MetS [42] prevalence among various races or ethnicities necessitate cautious adaptation and validation for broader applicability.

The other limitation of the nomogram is that prediction variables are multifaceted, comprising several modifiable factors that interact. Since changes in one variable can influence other variables, predicting outcomes based on single-variable changes can be intricate. However, enhancing these factors and embracing healthy behaviors can lead to improved metabolic status and potentially aid in obesity mitigation.

Despite the limitations, a comprehensive analysis was conducted using the data of all participants with obesity in South Korea over 4 years. The study and the developed nomogram can be applied generally to Koreans to predict the transition to MUO. For the next step, the research can be extended to people without obesity, finding associations between MHO and MUO and comparing risk factors among them. Since the nomogram can show intuitive results of predictions and be interpreted easily by any reader [13], it can be broadly used as a screening tool, leveraging national-level examination results. Coupled with advancements in digital health, telehealth, and wearable devices [43], the screening tool targeting metabolic status will play an essential role in directing appropriate interventions to those in need.

### Conclusions

The study explored the dynamics of young adults with obesity in South Korea, pinpointing factors that correlate with the transition from MHO to MUO and creating the predictive nomogram. In this study, the scoring arrangement for transitioning to MUO revealed that the major variables were triglycerides, waist circumference, HDL cholesterol, blood pressure, and fasting glucose. Although these risk factors were highlighted in previous studies [33-36], the significance of this study lies in the development of a nomogram that enables even the general population to easily and intuitively assess their likelihood of transitioning to MUO.

With the rising prevalence of obesity and increasing intervention strategies, our screening tool simplifies the prediction using easily identifiable factors for the general population. However, this study serves as a starting point, and further research should broaden models to include racial characteristics, thereby improving the relevance of the nomogram across diverse populations.

## Abbreviations

HDL: high-density lipoprotein
MetS: metabolic syndrome
MHO: metabolically healthy obesity
MUO: metabolically unhealthy obesity
NCD: noncommunicable disease
NHIS: National Health Insurance System
OR: odds ratio
WHO: World Health Organization

## Data Availability

The data sets generated and/or analyzed during this study are not publicly available because of the National Health Insurance Service regulation [44]. The customized database used in this study was formatted by the National Health Insurance Service in Korea, which strictly prohibits sharing the data publicly. Only permitted researchers can access the data set, and analysis can only be performed in the designated data center.
